# Are the Most Plastic Species the Most Abundant Ones? An Assessment Using a Fish Assemblage

**DOI:** 10.1371/journal.pone.0092446

**Published:** 2014-03-20

**Authors:** Nicolás Vidal, Natalia Zaldúa, Alejandro D'Anatro, Daniel E. Naya

**Affiliations:** Departamento de Ecología y Evolución - Facultad de Ciencias, Universidad de la República, Montevideo, Uruguay; University of Auckland, New Zealand

## Abstract

Few studies have evaluated phenotypic plasticity at the community level, considering, for example, plastic responses in an entire species assemblage. In addition, none of these studies have addressed the relationship between phenotypic plasticity and community structure. Within this context, here we assessed the magnitude of seasonal changes in digestive traits (seasonal flexibility), and of changes during short-term fasting (flexibility during fasting), occurring in an entire fish assemblage, comprising ten species, four trophic levels, and a 37-fold range in body mass. In addition, we analyzed the relationship between estimates of digestive flexibility and three basic assemblage structure attributes, i.e., species trophic position, body size, and relative abundance. We found that: (1) Seasonal digestive flexibility was not related with species trophic position or with body size; (2) Digestive flexibility during fasting tended to be inversely correlated with body size, as expected from scaling relationships; (3) Digestive flexibility, both seasonal and during fasting, was positively correlated with species relative abundance. In conclusion, the present study identified two trends in digestive flexibility in relation to assemblage structure, which represents an encouraging departure point in the search of general patterns in phenotypic plasticity at the local community scale.

## Introduction

Phenotypic plasticity refers to changes in organisms' traits due to changes in internal or external environmental conditions [1]. These adjustments could imply different kinds of phenotypic traits, from gene expression to life-history, and they usually constitute adaptations to cope with environmental variability [2]–[4]. In line with this, the evidence gathered over the last ten years suggests that the ability of different species to cope with current accelerated change in environmental conditions (i.e., the global environmental change) would be closely related to the amount of plasticity for fitness-related traits [5]–[10]. Thus, research aimed at determining the levels and limits of phenotypic plasticity in natural populations, as well as general patterns regarding phenotypic plasticity, are needed for both theoretical and practical reasons [11].

Empirical studies of phenotypic plasticity have analyzed plastic responses at different levels of biological organisation, ranging from those assessing a single population to those evaluating several, more or less unrelated, species at large geographic scales [12]–[15]. However, very few studies have evaluated phenotypic plasticity at the community level, considering, for example, plastic responses in an entire species assemblage (but see [16]–[17]). In addition, to our knowledge, none of these studies have addressed the relationship between phenotypic plasticity and community structure. This gap in current knowledge is relevant since the ultimate effect of all those factors associated with global environmental change will depend on processes and phenomena occurring at the local community scale [18]–[19].

Phenotypic flexibility –i.e., reversible changes in organisms' traits due to changes in environmental conditions [1]– in digestive traits comprises a classic model for the study of phenotypic plasticity in vertebrate animals. Experimental and field studies conducted over the last century clearly indicate that, among the physiological systems, the digestive system is one of the most reactive to change in environmental conditions [20]–[23]. On theoretical grounds, digestive flexibility –and particularly changes in the size of digestive organs (see [24]–[25])– is important for at least two reasons. First, it allows the animal to maximize energy and nutrients return from the specific diet that is being consumed. Second, it allows the animal to minimize the maintenance costs associated with one of the most expensive systems in terms of energy and protein metabolism [25]–[26]. In line with these ideas, experimental studies demonstrate that digestive flexibility can noticeably affect animals' performance (e.g., food assimilation), while comparative studies suggest that digestive flexibility has evolved by natural selection [25]–[28].

The present study aims to analyze the flexibility in digestive organ sizes for a fish assemblage, which comprises ten species, four trophic levels, and a 37-fold range in body mass. Specifically, our objectives were to: (1) Assess the magnitude of seasonal changes in digestive traits (seasonal digestive flexibility), and of changes during short-term fasting (digestive flexibility during fasting), occurring in ten fish species, and (2) Analyze the relationship between estimates of digestive flexibility and three assemblage structure attributes (species trophic position, body size, and relative abundance). We hypothesized that: (1) Given that species at lower trophic positions mainly consume resources that are abundant all year round (e.g., detritus, periphyton and algae), while species at higher trophic positions typically predate on resources whose abundance varies seasonally (e.g., insects, fish larvae and alevins), we predict a positive relationship between seasonal digestive flexibility and trophic position; (2) Given that energy needs for maintenance scale with body mass with an exponent lower than one (typically between 0.6 and 0.8), while digestive organ sizes scale isometrically with body mass –i.e., digestive processing capacity *per* unit of food to be processed increases with body size [25], [29]–, we predict a negative relationship between digestive flexibility during fasting and body size; (3) Given the potential impact of digestive flexibility on organisms' fitness, we predict that more flexible species, both in a seasonal and short-term basis, should have greater relative abundances than less flexible species.

## Materials and Methods

### Study area, species biology and sampling design

**Figure 1:**
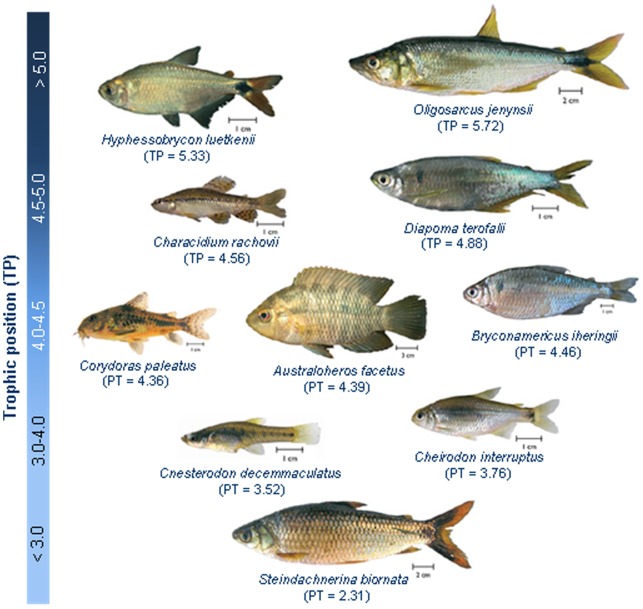
Species considered in our study, showing their trophic position according to stable isotopes.

The study area was located in “Arroyo de la Barra Falsa”, Punta Negra, Uruguay (34°53′S - 55°13′W), a small stream (maximum depth during winter *ca.* 1.5 m) with a mix of gravel, mud and stones in its bottom, and surrounded by a riparian forest of medium density. Individuals of each species were collected by an intensive use of differents trawl and cast nets (that cover the entire water column), set along *ca.* 100 m in the lower part of this stream. We collected ten fish species, which represent almost the entire fish assemblage in our study site (Fig. 1). Even though all these species could be considered omnivorous, in the sense that both vegetal and animal material have been found in their stomachs, there is a clear gradient in their trophic habits, from species that mainly consume detritus to those that mainly predate on invertebrates and other fish species (Table 1; [30]).

**Table 1:** List of the species investigated (sorted according to their trophic position), maximum standard length (StL_max_, in cm), trophic position (TP, estimated from stable isotopes), relative abundance (RA, 1: Rare, 2: Common, 3: Very common), and main dietary items consumed (Diet, taken from [30]).

Species	Order, Family	StL_max_	TP	RA	Diet
*Steindachnerina biornata*	Characiformes, Curimatidae	20	2.31	2	Detritus
*Cnesterodon decemmaculatus*	Cyprinodontiformes, Poeciliidae	4.5	3.52	3	Detritus, algae, zooplankton, and insect larvae
*Cheirodon interruptus*	Characiformes, Characidae	6.0	3.76	1	Detritus, algae, zooplankton, and insect larvae
*Corydoras paleatus*	Siluriformes, Callichthyidae	6.9	4.36	1	Periphyton, algae, zooplankton, and insect larvae
*Australoheros facetus*	Perciformes, Cichlidae	25.0	4.39	1	Periphyton, plant material, insects and small fish
*Bryconamericus iheringii*	Characiformes, Characidae	11.4	4.46	3	Periphyton and invertebrates
*Characidium rachovii*	Characiformes, Crenuchidae	5.0	4.56	2	Zooplankton and insects
*Diapoma terofali*	Characiformes, Characidae	7.0	4.88	2	Zooplankton and insects
*Hyphessobrycon luetkenii*	Characiformes, Characidae	6.9	5.33	3	Algae, plant material and insect larvae
*Oligosarcus jenynsii*	Characiformes, Characidae	20.0	5.72	2	Aquatic invertebrates and other fish species

Ten individuals of each species were collected during the Austral winter (four field expeditions in July-August 2010 and three in July 2011) and during the Austral summer (four field expeditions in January-February 2011 and two in January 2012). Fish were transported in a cooling box to the laboratory at Facultad de Ciencias – Universidad de la República (Montevideo), on the same day of their capture, and maintained in individual aquariums with dechlorinated tap water, at natural temperature and photoperiod during 24 h. These individuals were used to estimate seasonal flexibility in digestive organ sizes. During the Austral summer (four field expeditions in February 2012) we collected 19 additional individuals of each species, which were randomly assigned to one of two feeding groups: control (n = 10) or fasting (n = 9). Individuals in the control group were analyzed the same day of their capture, while individuals in the fasting group were analyzed after four days of fasting. We selected this short-term fasting period because we believe that it could be representative of real fasting periods occurring in nature. In addition, given the great variability in feeding habits of different species (see Table 1), we preferred to process control animals the day of their collection rather than feed them with an artificial diet for four days. During the fasting period fish were maintained in individual aquariums with dechlorinated tap water, at 26 ± 1 °C (the mean maximum temperature for January at our study site) and natural photoperiod (*ca.* 14L:8D). These individuals were used to estimate flexibility during fasting in digestive organ sizes.

The present study was conducted in the public space, outside protected areas, and involves non-endangered species for which specific collection permissions are not required. In addition, research conducted as part of this study conformed to national and institutional guidelines for research on live animals (approved by Comisión de Ética en Uso de Animales, Facultad de Ciencias, Universidad de la Repúlbica, Uruguay).

### Relative abundance estimations

Species relative abundance was estimated according to their occurrence in succesive field expeditions within each sampling event (July-August 2010: n = 4; July 2011: n = 3; January-February 2011: n = 4; January 2012: n = 2, February 2012: n = 4). Specifically, species were categorized as: (1) Very common, if the overall required number of specimens was collected during each field expedition in all sampling events; this group comprise *Cnesterodon decemmaculatus*, *Bryconamericus iheringii*, and *Hyphessobrycon luetkenii*; (2) Common, if the overall required number of specimens was completed only after successive field expeditions during, at least, one sampling event; this group comprise *Steindachnerina biornata*, *Characidium rachovii*, *Diapoma terofali*, and *Oligosarcus jenynsii*; and (3) Rare, if we could not complete the required number of specimens even after successive expeditions during, at least, one sampling event; this group comprise *Corydoras paleatus* (12 individuals collected for the seasonal analysis and 13 for the feeding experiment), *Cheirodon interruptus* (17 individudals collected for the feeding experiment) and *Australoheros facetus* (18 individuals collected for the feeding experiment). Note that, in despite of the low-resolution of our relative abundance estimations, obtained results are in good agreement with density estimations existing for several Uruguayan streams with similar characteristics to those of our study site (see Discussion).

### Morphometric determinations

Fish were anaesthetized with 2-phenoxyethanol, and body mass (m_b_) was measured with an electronic balance (± 0.0001 g; AND HR-200; Japan) and standard length (StL) with a plastic ruler (0.1 cm). Then, animals were sacrificed by spinal transection, and their intestine (including pyloric caeca) and liver were removed. The intestine was carefully dissected to avoid tissue stretching and its length was measured with a digital caliper (± 0.01 mm; Litz Professional; Germany). Intestine and pyloric caeca (hereafter just intestine) were completely emptied of material, rinsed with a 0.9% NaCl solution, carefully dried with paper towels, and weighed (± 0.0001 g). Finally, intestine, liver and animal's carcass, were dried in an oven (Termaks, Series 2000; Norway) at 60°C until constant weight was reached (7 d), and then weighed individually (± 0.0001 g).

### Trophic position estimations

Five individuals of each species, collected during summer 2012, were used to determine *δ* ^13^C_standard_ and *δ* ^15^N_standard_ values. At the same time, we collected five individuals of a filter-feeding bivalve (*Corbicula fluminea*), to estimate the base level of the pelagic component of the food web, and five individuals of an herbivorous snail (*Pomacea canaliculata*), to estimate the base level of the littoral component of the food web. Muscle tissue samples were taken from fish and molluscs, and dried in an oven at 60°C for 48 h, packed, and sent to Centro de Energia Nuclear na Agricultura – Universidad de Sao Paulo (Brazil) for isotopic determination. The trophic position of each species (Fig. 1, Table 1) was then estimated using the following equation [31]:



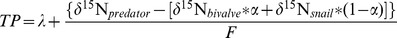
,where λ is the trophic level of consumers at the base of the food web (in this case λ = 2 because snails and bivalves are primary consumers), *δ* ^15^N_predator_ is the nitrogen signature of the consumer being evaluated, α is the proportion of carbon derived from the pelagic food web base:



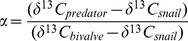
,and F is the *per* trophic level fractionation of nitrogen (3.4‰; [32]). Lipids were not removed from muscle samples because protocols used to remove lipids may adversely affect nitrogen isotope integrity (see [33]). In addition, given that analyses done with raw data yielded virtually the same results as analysis done with data normalized for differences in lipid content between species (following [33]) –agreeing with the fact that C:N was lower than 3.5 for nine of the ten fish species analyzed– herein we only present those results obtained with raw isotopic data.

### Data analysis

We estimated phenotypic flexibility (*sensu lato*; see [34]) for each morphometric variable of each species through the Hedges' difference (d); that is, the difference between winter and summer (or control and fasting) body size-adjusted means, expressed in units of pooled standard deviation and corrected for small sample bias (see [35]–[37]). Seasonal digestive flexibility (d_s_) was calculated as the absolute seasonal change in organ sizes, regardless the season in which larger values occurred. Digestive flexibility during fasting (d_f_) was calculated in such a way that positive values indicate larger organ sizes in the control group. We evaluated the relationship between each measure of flexibility for each morphometric variable and independent variables (e.g., species body mass, standard length, trophic position, and relative abundance), through random-effect meta-analytical models with continuous predictor variables (see [35]-[36]). All these analyses were conducted using the software Metawin version 2.0 [35]. Statistical significance was established at the 0.05 level.

We did not attempt to use phylogenetically informed analyses, because continuous traits evaluated over large temporal scales (e.g., tens of millons of years) are not expected to be phylogenetically constrained [38]. In line with this, experiments conducted in rodents indicated that intestinal weight can evolve very fast, changing as much as 35% after forty generations of artificial selection [39].

## Results

**Figure 2:**
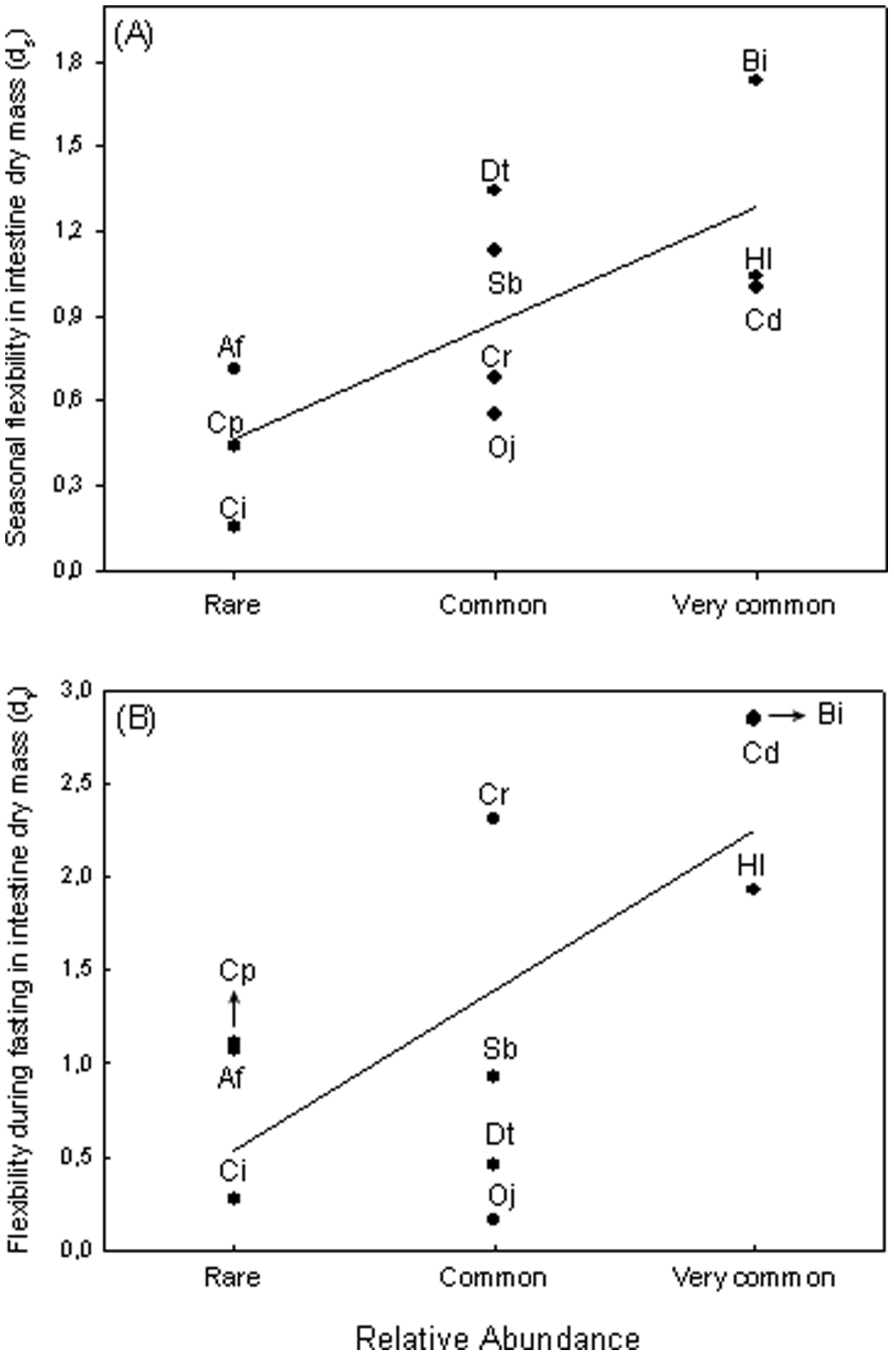
Relationships between: (A) Species relative abundance and seasonal flexibility in intestine dry mass, and (B) Species relative abundance and flexibility during fasting in intestine dry mass. Species abbreviations: Sb =  *Steindachnerina biornata*; Cd =  *Cnesterodon decemmaculatus*; Ci =  *Cheirodon interruptus*; Cp =  *Corydoras paleatus*, Af =  *Australoheros facetus*; Bi =  *Bryconamericus iheringii*; Cr =  *Characidium rachovii*; Dt =  *Diapoma terofali*; Hl =  *Hyphessobrycon luetkenii*; Oj =  *Oligosarcus jenynsii*.

Data on body size and morphometric variables for each species in each season are provided in Table 2 (and in the Data S1), while data for each species in each feeding group are given in Table 3 (and in the Data S1). The only significant correlation between independent variables was the one between standard length and body mass (Table 4), allowing independent testing of our three predictions. In this sense, we found that: (1) Species trophic position was not correlated with seasonal flexibility for any morphometric variable (Table 5); (2) Species standard length was negatively correlated with flexibility during fasting for intestine and liver dry masses, even though both correlations only reach a marginal probability value (Table 5); (3) Species relative abundance was positively correlated with seasonal flexibility, and also with flexibility during fasting, for intestine dry mass (Table 5; Fig. 2); in addition, relative abundance was positively correlated with flexibility during fasting for intestinal length, but this correlation only reached a marginal probability value (Table 5).

**Table 2:** Body size and morphometric variables for the species investigated in each season.

Species	StL (cm)	m_b_ (g)	IL (cm)	IM (g)	LM (g)
*S. biornata*					
Summer (n = 10)	7.8 (0.5)	13.9 (1.9)	72.4 (6.9)	0.0788 (0.0098)	0.0309 (0.0049)
Winter (n = 10)	7.1 (0.6)	10.9 (3.7)	58.5 (8.4)	0.0426 (0.0096)	0.0210 (0.0055)
*C. descemmaculatus*					
Summer (n = 10)	2.8 (0.1)	0.36 (0.03)	5.8 (0.4)	0.0017 (0.0002)	0.0014 (0.0004)
Winter (n = 10)	2.5 (0.1)	0.30 (0.03)	5.3 (0.3)	0.0024 (0.0002)	0.0014 (0.0001)
*C. interruptus*					
Summer (n = 10)	4.3 (0.1)	1.7 (0.2)	3.5 (0.1)	0.0062 (0.0010)	0.0043 (0.0005)
Winter (n = 10)	4.1 (0.2)	1.5 (0.2)	3.2 (0.2)	0.0058 (0.0007)	0.0050 (0.0009)
*C. paleatus*					
Summer (n = 8)	3.7 (0.1)	1.8 (0.2)	4.0 (0.2)	0.0111 (0.0041)	0.0071 (0.0014)
Winter (n = 4)	4.0 (0.3)	2.0 (0.4)	3.6 (0.4)	0.0064 (0.0011)	0.0095 (0.0027)
*A. fascetus*					
Summer (n = 10)	5.0 (0.5)	6.6 (1.3)	3.7 (0.5)	0.0155 (0.0035)	0.0210 (0.0050)
Winter (n = 10)	4.1 (0.2)	2.8 (0.4)	4.0 (0.2)	0.0094 (0.0010)	0.0189 (0.0026)
*B. iheringii*					
Summer (n = 10)	5.2 (0.1)	3.0 (0.2)	4.1 (0.2)	0.0167 (0.0012)	0.0113 (0.0006)
Winter (n = 10)	5.5 (0.2)	3.7 (0.5)	4.5 (0.2)	0.0285 (0.0003)	0.0341 (0.0152)
*C. rachovi*					
Summer (n = 10)	3.9 (0.1)	1.0 (0.1)	1.3 (0.1)	0.0025 (0.0001)	0.0032 (0.0002)
Winter (n = 10)	3.9 (0.1)	1.0 (0.1)	1.4 (0.1)	0.0030 (0.0003)	0.0034 (0.0003)
*D. terofali*					
Summer (n = 10)	6.1 (0.1)	3.9 (0.3)	2.2 (0.04)	0.0072 (0.0005)	0.0070 (0.0010)
Winter (n = 10)	5.9 (0.1)	3.8 (0.3)	2.5 (0.13)	0.0104 (0.0009)	0.0087 (0.0009)
*H. luetkenii*					
Summer (n = 10)	5.0 (0.1)	3.2 (0.3)	3.7 (0.2)	0.0111 (0.0014)	0.0141 (0.0028)
Winter (n = 10)	5.2 (0.1)	3.1 (0.3)	4.0 (0.2)	0.0212 (0.0039)	0.0128 (0.0017)
*O. jenninsi*					
Summer (n = 10)	8.0 (1.1)	11.3 (4.6)	3.0 (0.4)	0.0189 (0.0084)	0.0238 (0.0094)
Winter (n = 10)	9.2 (0.5)	12.2 (2.5)	3.8 (0.7)	0.0319 (0.0057)	0.0305 (0.0059)

StL =  standard length, m_b_ =  body mass, IL =  intestine length, IM =  intestine and pyloric ceca dry mass, LM =  liver dry mass. Data presented are absolute means ± 1 SEM (StL and m_b_) or least squares adjusted means ± 1 SEM (using StL as covariate for IL, and carcass dry mass as covariate for IM and LM).

**Table 3:** Body size and morphometric variables for the species investigated in each feeding group.

Species	StL (cm)	m_b_ (g)	IL (cm)	IM (g)	LM (g)
*S. biornata*					
Control (n = 10)	8.1 (0.5)	15.9 (2.5)	95.7 (9.7)	0.1047 (0.0192)	0.0335 (0.0062)
Fasting (n = 9)	8.4 (0.1)	14.3 (0.6)	56.6 (2.9)	0.0562 (0.0112)	0.0288 (0.0033)
*C. descemmaculatus*					
Control (n = 10)	3.4 (0.1)	0.68 (0.05)	8.1 (0.2)	0.0038 (0.0002)	0.0044 (0.0003)
Fasting (n = 9)	3.0 (0.1)	0.46 (0.07)	5.8 (0.2)	0.0020 (0.0001)	0.0018 (0.0003)
*C. interruptus*					
Control (n = 10)	4.1 (0.1)	1.6 (0.2)	3.9 (0.3)	0.0068 (0.0013)	0.0050 (0.0007)
Fasting (n = 7)	4.1 (0.3)	1.4 (0.5)	3.8 (0.3)	0.0056 (0.0014)	0.0053 (0.0018)
*C. paleatus*					
Control (n = 5)	3.9 (0.3)	2.5 (0.5)	4.0 (0.4)	0.0133 (0.0028)	0.0099 (0.0024)
Fasting (n = 8)	4.3 (0.2)	3.1 (0.4)	3.8 (0.2)	0.0080 (0.0010)	0.0082 (0.0017)
*A. fascetus*					
Control (n = 10)	6.7 (0.3)	12.2 (1.4)	6.0 (0.3)	0.0319 (0.0033)	0.0449 (0.0067)
Fasting (n = 8)	6.5 (0.3)	11.3 (1.9)	5.1 (0.5)	0.0212 (0.0029)	0.0306 (0.0053)
*B. iheringii*					
Control (n = 10)	5.4 (0.1)	3.3 (0.2)	4.0 (0.2)	0.0185 (0.0011)	0.0051 (0.0006)
Fasting (n = 9)	5.0 (0.2)	2.5 (0.2)	3.4 (0.3)	0.0105 (0.0004)	0.0047 (0.0004)
*C. rachovi*					
Summer (n = 10)	4.2 (0.1)	1.4 (0.1)	1.1 (0.1)	0.0029 (0.0002)	0.0036 (0.0005)
Winter (n = 9)	3.9 (0.1)	1.1 (0.1)	1.2 (0.1)	0.0018 (0.0001)	0.0028 (0.0002)
*D. terofali*					
Control (n = 10)	5.9 (0.2)	3.7 (0.4)	1.8 (0.1)	0.0049 (0.0006)	0.0051 (0.0011)
Fasting (n = 9)	4.3 (0.3)	1.4 (0.2)	1.7 (0.1)	0.0042 (0.0005)	0.0034 (0.0005)
*H. luetkenii*					
Control (n = 10)	5.0 (0.1)	2.8 (0.2)	3.4 (0.2)	0.0102 (0.0010)	0.0067 (0.0006)
Fasting (n = 9)	4.9 (0.1)	2.5 (0.2)	2.8 (0.1)	0.0050 (0.0007)	0.0072 (0.0009)
*O. jenninsi*					
Control (n = 10)	11.7 (0.7)	24.0 (4.3)	3.4 (0.4)	0.0489 (0.0189)	0.0299 (0.0115)
Fasting (n = 9)	7.7 (1.0)	7.7 (2.2)	3.3 (0.3)	0.0415 (0.0034)	0.0329 (0.0039)

StL =  standard length, m_b_ =  body mass, IL =  intestine length, IM =  intestine and pyloric ceca dry mass, LM =  liver dry mass. Data presented are absolute means ± 1 SEM (StL and m_b_) or least squares adjusted means ± 1 SEM (using StL as covariate for IL, and carcass dry mass as covariate for IM and LM).

**Table 4:** Pearson product-moment correlation coefficients (and associated P-values) for the relationships between independent variables.

Independent variables	r	P
Standard length vs. Body mass	***0.92***	***0.0001***
Standard length vs. Trophic position	0.18	0.62
Standard length vs. Relative abundance	0.03	0.93
Body mass vs. Trophic position	−0.10	0.79
Body mass vs. Relative abundance	−0.05	0.88
Trophic position vs. Relative abundance	0.11	0.76

**Table 5:** Pearson product-moment correlations (and associated results of meta-analytical models) for the correlation between flexibility in morphometric variables and standard length, body mass, trophic position, and relative abundance.

		Seasonal flexibility (d_s_)			Flexibility during fasting (d_f_)		
		r	*X^2^*	P	r	*X^2^*	P
**Standard length**	Intestine length	0.32	0.22	0.64	−0.29	0.49	0.48
	Intestine mass	0.17	0.15	0.70	−0.55	3.67	0.06
	Liver mass	0.41	0.39	0.53	−0.56	3.24	0.07
**Body mass**	Intestine length	0.09	0.02	0.88	−0.09	0.01	0.92
	Intestine mass	0.08	0.04	0.84	−0.51	2.70	0.10
	Liver mass	0.31	0.21	0.64	−0.39	1.37	0.24
**Trophic position**	Intestine length	0.08	0.01	0.92	−0.48	2.79	0.10
	Intestine mass	−0.05	0.02	0.88	−0.10	0.09	0.77
	Liver mass	−0.21	0.08	0.77	−0.35	1.07	0.30
**Relative abundance**	Intestine length	0.10	0.02	0.88	0.54	3.26	0.07
	Intestine mass	*0.72*	*3.97*	*0.05*	*0.69*	*6.04*	*0.01*
	Liver mass	−0.10	0.01	0.93	0.29	0.46	0.50

d.f. = 8 in all the cases.

## Discussion

The present paper comprises one of the few studies analyzing phenotypic plasticity in an entire animal assemblage [16]–[17], and –to the best of our knowledge– it is the first one aimed to identify general patterns in phenotypic plasticity in relation to basic community structure attributes. Even though we worked with an assemblage composed of relatively few species, we followed a highly standardized methodology throughout the study, reducing residual variation in the estimations of digestive flexibility. In addition, we worked in a natural system where basic assemblage attributes were not correlated among them, allowing to test our predictions in a fairly independent fashion. This way, we were able to identify two interesting trends in digestive flexibility, which will comprise the focus of the following discussion.

### Digestive flexibility during fasting and body size

Based on scaling relationships of maintenance costs and organ sizes, a negative relationship between flexibility in digestive organ sizes and body size has been proposed [25], [29], [40], but –to our knowledge– not yet empirically tested. Here we found some support for this prediction, since there seemed to be a negative correlation between flexibility during fasting and standard length for intestine and liver masses. Even though both correlations were weak, predictions based on scaling factors usually need variation over several orders of magnitude in body size so as to be detected [41], which is not the case of our study. On the other hand, it is important to note that we evaluated digestive flexibility during fasting, i.e. using an experimental condition during which animals rely on their energetic reserves. Given that energy storage organs also scale isometrically with body mass, obtained results could be due, at least in part, to the fact that bigger species have larger absolute and relative (i.e., *per* unit of metabolic mass) energy reserves than smaller species do. Consequently, larger species can reduce the size of their digestive organs at a slower rate than smaller species, which would be beneficial because a reduced gut may represent an important penalty when feeding is resumed. In any case, this additional interpretation concurs with, but does not replace, the above mentioned theoretical reasons for expecting a negative correlation between digestive flexibility and body size.

### Digestive flexibility and relative abundance

**Figure 3:**
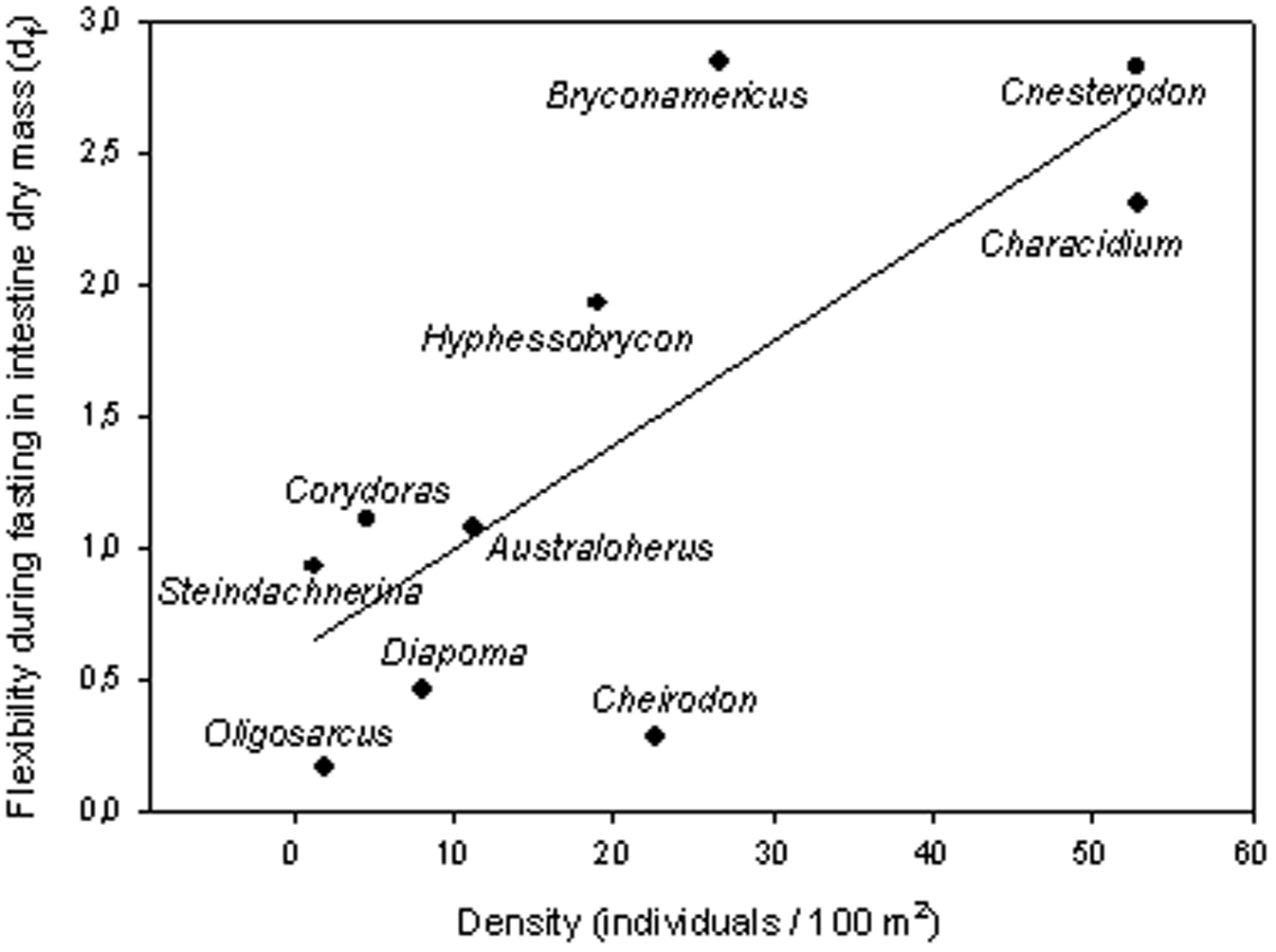
Relationship between average fish density at the genus level, estimated from seven streams of similar characteristics to those of our study site (see main text), and flexibility during fasting in intestine dry mass for each species of this genus.

Phenotypic plasticity in fitness-related traits has been broadly investigated in the context of biological invasions [42]–[43]. However, few attempts have been made to connect phenotypic plasticity in key traits with species abundance in their native communities (e.g., [17]). Here, we found that digestive flexibility, both seasonal and during fasting, was positively related with species relative abundance. Even though we used a broad quantification of relative abundances, there are at least three facts that support the reported pattern. First, the two most plastic species (*B. iheringi* and *C. decemmaculatus*) were also the most abundant species in a survey of seven Uruguayan streams, with similar characteristics to those of our study site [44]. Second, using density values reported by these authors (in their Fig. 3), we found a significant positive correlation between species density and flexibility during fasting for intestine dry mass (r = 0.64, *X^2^* = 4.90, P = 0.03), liver dry mass (r = 0.77, *X^2^* = 7.53, P = 0.01), and, marginally, for intestinal length (r = 0.57, *X^2^* = 2.72, P = 0.09). Third, for the case of intestine dry mass –i.e., the most relevant variable regarding food processing capacities– this correlation is even noticeable when densities are considered at the genus level (r = 0.75, *X^2^* = 9.95, P = 0.002; Fig. 3), that is, after solving the problem of local substitution of species within the same genus from one stream to another. Thus, the positive relationship between digestive flexibility and species relative abundance probably is not an artifact of our broad estimation of this last variable. A remaining point to be solved, however, is to what extent greater flexibility is the cause behind higher abundances, or if the positive correlation between these two variables is just due to a link with another variable, typically body size (e.g.., smaller species are the most flexible due to scaling factors and also the most abundant due to resource partitioning among species). Even though relative abundance was not correlated with body size in our data set, suggesting that greater flexibility could be the cause of higher abundances, we believe that this relevant issue deserves deeper exploration.

## Conclusions

Phenotypic plasticity has been suggested as the main mechanism for species persistence under a global environmental change scenario [5]–[10]. However, models aimed to predict the effect of climatic change on future species distribution do not take into account differences in plastic responses among species ([45], but see [46]). This is mainly because we still do not have data on plasticity for fitness-related traits for most of the species to be modelled. An encouraging pathway to fill this gap is the identification of general patterns in phenotypic plasticity at both local and global scales, which could be easily incorporated into the models [11]. Here we were able to identify two interesting trends in digestive flexibility in relation to assemblage structure attributes, comprising an important starting point in the search of general patterns in phenotypic plasticity at a local community scale. However, several important issues should be addressed in further studies. In particular, to disentangle if the observed positive correlation between phenotypic plasticity and species abundance is just a by-product of the correlation between both variables and body size is very relevant in the context of the environmental global change. This is because small sized species usually have a greater microevolutionary potential –due to larger population sizes and shorter generation times– than larger species, and thus, phenotypic plasticity probably is more important for populations persistance in the later species than in the former ones (see [47]).

## Supporting Information

Data S1 **Raw data on body size and morphometric variables for the species investigated in each season and each feeding group.**
(XLSX)
